# Enriched Fresh Noodles Incorporating Chestnut Starch–Resveratrol Complexes: Quality, Structural Properties and Predicted Glycemic Index

**DOI:** 10.3390/foods15091547

**Published:** 2026-04-29

**Authors:** Lu Li, Yawei Xu, Yunfei Huang, Yufan Wu, Chunmei Li

**Affiliations:** 1 College of Food Science and Technology, Huazhong Agricultural University, Wuhan 430070, China; li1997@webmail.hzau.edu.cn (L.L.);; 2 Key Laboratory of Environment Correlative Food Science, Ministry of Education, Huazhong Agricultural University, Wuhan 430070, China

**Keywords:** fresh noodles, starch–polyphenol complex, extrusion, digestibility, glycemic index

## Abstract

Starch–polyphenol complexes have attracted increasing attention as functional ingredients for improving the structural stability and reducing the glycemic potential of starch-based foods, yet their application in extruded fresh noodles remains insufficiently understood. In this study, chestnut starch–resveratrol complexes prepared by heat-moisture synergistic recrystallization treatment (CS-HMRT-Res) were incorporated into extruded fresh noodles, and their quality, structural characteristics, digestibility, and glycemic response were systematically evaluated. Compared with commercial wheat-based Regan noodles, CS-HMRT-Res noodles exhibited enhanced cooking stability (lower swelling and leaching) and improved texture (hardness, chewiness, tensile strength), with a markedly lower total color difference after cooking (ΔE = 1.8 vs. 6.5). SEM, FTIR and XRD indicated a more compact and ordered network; the relative crystallinity of cooked noodles increased to approximately 30.8%. In in vitro digestion, CS-HMRT-Res showed the lowest starch hydrolysis extent at 180 min (45.92%) and yielded a low predicted glycemic index of 53.35, compared with 70.65 for Regan noodles. Consistently, gavage studies in mice confirmed that HMRT-Res-chestnut starch produced the lowest postprandial blood glucose increment response (4.31 mmol/L). Molecular dynamics simulations further suggested that resveratrol could competitively occupy the α-amylase binding cavity and reduce starch accessibility to the enzyme. Overall, CS-HMRT-Res improved processing quality, structural integrity, and reduced glycemic potential, offering a structure-function framework for designing low-GI products.

## 1. Introduction

Wheat noodles have been consumed for millennia in Asia and remain a primary carbohydrate source, valued for their satiety, convenience, and cultural significance. From a materials perspective, noodle quality and metabolic response are governed by the organization of starch within a protein-rich matrix. Starch structure (granular integrity, molecular order, and crystalline organization) controls water uptake, swelling, and cooking loss, while also dictating enzyme accessibility during digestion [1]. At the same time, non-starch constituents (particularly gluten proteins and lipids) and their intermolecular interactions further regulate rheology, microstructure, and digestion behavior. Conventional fresh noodle production, typically involving sequential steps such as mixing, sheeting, and cutting, often yield products with rapid starch hydrolysis and substantial solid leaching during cooking [2], which jointly contribute to high postprandial glycemic responses and reduced cooking tolerance. These limitations are increasingly relevant as the global burden of obesity and type 2 diabetes continues to rise, creating demand for staple foods that retain desirable texture while delivering a lower glycemic impact [3].

Extrusion technology offers an attractive manufacturing alternative, integrating mixing, thermal treatment, and forming into a continuous operation, thereby improving efficiency and product consistency [4]. Although extrusion is well-established for pasta and snack foods, its application in Chinese-style fresh noodles is still developing [5]. Recent studies have highlighted that extrusion-modified ingredients can effectively improve enriched noodle quality. Zhu et al. [2] found that extruded highland barley flour significantly influenced wheat dough thermomechanical behavior and improved the quality of fresh wet noodles. Moreover, Wang et al. [6] demonstrated that extrusion in phenolic-enriched noodle systems involves concurrent starch gelatinization/degradation and phenolic–starch complexation, which together affect product structure and starch digestibility. These findings support the importance of extrusion-induced structural regulation in the development of functional noodle products. Concurrently, starch–polyphenol complexes have gained attention as functional ingredients that can reduce starch digestibility through physical encapsulation, enhanced molecular ordering, and formation of enzyme-resistant helical inclusion complexes [7,8,9]. Notably, complexation behavior can be strongly influenced by thermo-mechanical processing, including extrusion, which may both promote complex formation and affect polyphenol release and distribution [10]. Tang et al. [11] successfully fabricated extruded buckwheat noodles by incorporating 3% epigallocatechin gallate and caffeic acid; however, the reduction in the predicted glycemic index was moderate, decreasing only from 75.39 to 63.38.

Among other potential ingredients, chestnut starch (CS), characterized by its unique C-type crystalline structure [12,13], and resveratrol (Res), a natural polyphenol, can participate in noncovalent interactions with starch chains and proteins and has also been reported to inhibit α-amylase [14,15,16]. Our previous research found that CS and Res can form a complex under the heat-moisture synergistic recrystallization treatment (CS-HMRT-Res) [17]. Nevertheless, the behavior of CS-HMRT-Res complexes during extrusion, their subsequent influence on the structural organization of the fresh noodle matrix, and the underlying mechanisms regulating digestibility remain poorly understood.

Therefore, this study aimed to: (1) develop extruded fresh noodles fortified with CS-HMRT-Res complexes and evaluate cooking, textural, sensory, and structural properties; (2) assess in vitro starch digestion kinetics and in vivo glycemic response; and (3) elucidate the molecular interaction mechanisms among Res, starch chains, and α-amylase through molecular dynamics simulations. By bridging processing, structural, and nutritional functionality, this work seeks to establish a comprehensive framework for designing novel noodles with improved quality and reduced glycemic impact.

## 2. Materials and Methods

### 2.1. Materials

Regan noodles and buckwheat noodles were purchased from Yuehuoli Supermarket (Wuhan, China). Commercial wheat flour (12.2% protein, 8.5% moisture) was obtained from Yihai Kerry Oils and Grains Industries Co., Ltd., Wuhan, China. Chestnut starch was extracted by the alkaline extraction method, as previously described by Li et al. [17]. Briefly, chestnut starch was extracted with 0.2% NaOH at a ratio of 1:2 (*w*/*v*) and contained 9.15% moisture, 0.15% protein and 0.20% lipid, as determined by AOAC methods. Analytical grade chemicals were used unless otherwise stated.

### 2.2. Preparation of HMRT-Chestnut Starch and HMRT-Res-Chestnut Starch

The preparation followed a previously described method [18]. Briefly, chestnut starch was premixed with Res (0%, 10% *w*/*w*, based on starch weight), adjusted to 20% moisture content, heated at 100 °C for 4 h, cooled at 4 °C for 4 h, and then washed and dried to obtain modified starch, which was designated as HMRT-Chestnut starch and HMRT-Res-Chestnut starch, respectively.

### 2.3. Extrusion Processing and Noodle Preparation

According to the preliminary experiment, noodle dough was formulated by blending wheat flour and modified starch at 57:43 (*w*/*w*, dry basis), with 0.8% (*w*/*w*) edible salt and 0.2% (*w*/*w*) edible alkali. Total moisture content was adjusted to 55% (*w*/*w*) based on the total dry weight of the solid ingredients. The extrusion process was carried out using a twin-screw extruder with different pitch (ZE-16, Weisite Technology Co., Ltd., Suzhou, China) under the following parameters: the screw length to diameter ratio (L/D) was 40:1; the die hole diameter was 3 mm; the four-stage temperatures were set at 50, 80, 108, and 90 °C (feeding, mixing, melting and die zone, respectively); and the screw speed was 136 rpm. The resulting extruded noodles prepared with untreated chestnut starch, HMRT-Chestnut starch and HMRT-Res-Chestnut starch were labeled CS, CS-HMRT and CS-HMRT-Res, respectively. After extrusion, fresh noodles were sealed in polyethylene bags and stored at 4 °C for 6 h. All analyses were performed on the same day after preparation.

### 2.4. Cooking Properties Determination

Water absorption was tested in triplicate according to AACC 56–40. Briefly, fresh noodles (5.00 g, M1) were cooked in 100 g boiling water for the optimum time, rinsed under running water for 10 s, drained, and re-weighed (M2). The optimum cooking time was determined as the time required for the disappearance of the white core in the noodle strand when gently squeezed between two glass plates, following the standard procedure. The water absorption rate was calculated using Equation (1). The diameter of noodles before and after cooking was measured using Vernier caliper to determine the diameter expansion rate. The cooking broth was made up to 100 mL, and its transmittance T (%) was measured at 600 nm using deionized water as a blank control.

(1) $$Water\;absorption\;rate\;\left(\%\right)=\frac{M2-M1}{M1\times (1-M)}\times 100\%$$

M is the moisture content of fresh noodles; M1 is the weight of fresh noodles; and M2 is the weight of the cooked fresh wet noodles after cooling and draining.

### 2.5. Color Measurement

Color parameters (L*, a*, b*) were measured using Ultra Scan VIS Colorimeter (Hunter Lab, Reston, VA, USA). L* indicated lightness, a* denoted the red-green component, and b* represented the yellow-blue component. Each sample was measured in triplicate and three strands analyzed per sample. The total color difference (ΔE) was calculated using Equation (2):

(2) $$\Delta E=\sqrt{{\left(100-L^{*}\right)}^{2}+a^{*2}+b^{*2}}$$

### 2.6. Texture and Tensile Properties

Noodles were cooked for 5 min, rinsed under cold water for 30 s, and blotted dry with absorbent paper prior to analysis. Texture profile analysis was performed using a TA-XT Plus texture analyzer (Stable Micro Systems Ltd., Godalming, UK) equipped with a P/36R probe. Test conditions were as follows: pre-test speed 2 mm/s, test speed 1 mm/s, post-test speed 2 mm/s, target strain 70%, and a 5 s pause between compressions. Each sample was measured in triplicate and three strands analyzed per sample. Parameters such as hardness, springiness, cohesiveness, gumminess, chewiness, and resilience were recorded from the test curves.

Tensile properties were determined using an A/SPR probe. The initial gap between the upper and lower fixtures was set to 8 mm. The test protocol involved an initial stretch of 10 mm, followed by a continuous extension at a speed of 2 mm/s until the noodle strand ruptured. The pre-test and post-test speeds were both 1 mm/s and 2 mm/s, respectively, with a trigger force of 5 g [19]. One strand was analyzed per replicate, with a total of three strands per formulation.

### 2.7. Electronic Nose Measurement

Cooked noodle aroma profiles were assessed using an electronic nose (PEN3, Airsense Analytics GmbH, Schwerin, Germany). Noodles (3.0 g) were boiled in 50 mL water for 5 min, cooled to room temperature, and transferred to a 20 mL sealed headspace vial. The samples were equilibrated at room temperature for 30 min, then incubated at 80 °C for 20 min to enrich volatile compounds. Data were collected for 120 s, followed by a 120 s sensor purge between runs.

### 2.8. Sensory Evaluation

Sensory quality was evaluated by 20 trained panelists (10 males and 10 females, ranging from 20 to 30 years old) according to Chinese Standard GB/T 35875-2018 [20]. Assessments were conducted in individual booths to ensure independent judgment. Each sample was scored for color (20), visual appearance (10), oral comfort (20), chewiness and elasticity (30), surface smoothness (15), and taste and aroma (5). All procedures were approved by the Animal Welfare and Ethics Committee of Huazhong Agriculture University (No. HZAUHU-2025-0015. Date: 12 January 2025).

### 2.9. Scanning Electron Microscopy (SEM)

The uncooked and cooked noodles were freeze-dried (LGJ-10, Beijing Songyuanhuaxing Technology Development Co., Ltd., Beijing, China). Samples were mounted on conductive adhesive tape, sputter coated with gold for 30 s, and imaged using SEM (SU3800, Hitachi Science & Technology Beijing Co., Tokyo, Japan) at 500× magnification.

### 2.10. Fourier Transform Infrared Spectroscopy (FTIR)

FTIR spectra were recorded using a TENSOR II spectrometer (Bruker Optics, Leipzig*,* Germany). Measurements were conducted in the range of 4000–400 cm^−1^ with a spectral resolution of 4 cm^−1^, and each spectrum was obtained by averaging 32 scans.

### 2.11. X-Ray Diffraction (XRD) Measurement

The crystalline structure was analyzed using an X-ray diffractometer (D8 ADVANCE, Bruker AXS GmbH, Karlsruhe, Germany) with Cu Kα radiation. Diffraction patterns were collected in continuous scan mode with a scan rate of 4 °/min over the 2θ range of 5–45°, employing a step size of 0.02°. Data were processed using MDI Jade 6.0 software (Materials Data Inc., Livermore, CA, USA).

### 2.12. In Vivo Glycemic Response

The animal experiment followed Zheng et al. [21]. A total of 40 KM male mice (4–6 weeks) were acclimatized for one week under sterile, standard conditions (12 h light/dark, 25 ± 2 °C) with free access to food and water. Mice were then randomly divided into five groups (n = 8). An amount of 1.8 g of starch (Wheat starch, Chestnut starch, HMRT-Chestnut starch, HMRT-Res-Chestnut starch) was mixed with 18 mL water, gelatinized at 95 °C for 15 min, and cooled to 37 °C. A glucose reference solution (2 mg/g body weight, 200 mg/mL) was administered by gavage to the glucose group. Starch paste was administered at 0.1 mL per 10 g body weight. Blood glucose was measured from tail vein blood using a glucometer (GA-3, Sinocare Inc., Changsha, China) at 0, 15, 30, 45, 60, 90, and 120 min after intervention. All procedures were approved by the Animal Welfare and Ethics Committee of Huazhong Agriculture University (No. HZAUMO-2025-0019. Date: 17 February 2025).

### 2.13. In Vitro Digestion Kinetics and Predicted Glycemic Index (pGI)

In vitro digestion was simulated as follows: noodle samples (1.5 ± 0.05 g) were first incubated with 5 mL of pepsin solution (2500 U/mL) at 37 °C for 30 min with shaking (200 rpm) to simulate the gastric phase. The reaction was then neutralized with 5 mL of NaOH (0.01 M). The intestinal phase was initiated by adding 15 mL of phosphate buffer (0.1 M, pH 6.8), 5 mL of α-amylase (250 U/mL), 0.1 mL of amyloglucosidase (3000 U/mL), and 1.0 mL of trypsin (1 mg/mL). This mixture was shaken at 37 °C and 180 rpm. At scheduled intervals (0, 20, 30, 60, 90, 120, 150 and 180 min), 0.2 mL aliquots were withdrawn, enzymatically inactivated with 75% ethanol (3.8 mL), and centrifuged (8000× *g*, 10 min). The glucose content in the supernatant was quantified with a glucose assay kit to determine the digestion kinetics. The hydrolysis index (HI) was calculated from the area under the hydrolysis curve, and pGI was calculated using Equation (3) [22]:

(3) pGI=0.862HI+8.1981 r=0.877,P<0.0001

### 2.14. Molecular Dynamics Simulation

The molecular dynamics simulations (MD) were performed following Pan et al. [23]. Periodic boundary conditions were applied in all three spatial dimensions. The Newtonian equations of motion were integrated using the Leapfrog algorithm. During NPT simulations, the pressure was controlled isotropically at 1 bar using the Berendsen barostat, and the V-rescale thermostat was used to keep the temperature constant at 310 K. Electrostatic interactions were determined using the Particle-Mesh-Ewald (PME) method, and a cutoff distance of 1.2 nm was applied for short-range van der Waals interactions. Interaction plots were visualized using Discovery Studio 2021 software.

### 2.15. Statistical Analyses

All experiments were performed in triplicate with independent samples. Data were analyzed using SPSS 27 (IBM Corp., Armonk, NY, USA). Differences were considered significant at *p* < 0.05 (Duncan’s multiple range test). Graphs were generated using Origin 2021 (Origin Lab Inc., Northampton, MA, USA), and error bars represented standard deviations.

## 3. Results and Discussion

### 3.1. Quality Attributes

#### 3.1.1. Cooking Characteristics

**Figure 1:**
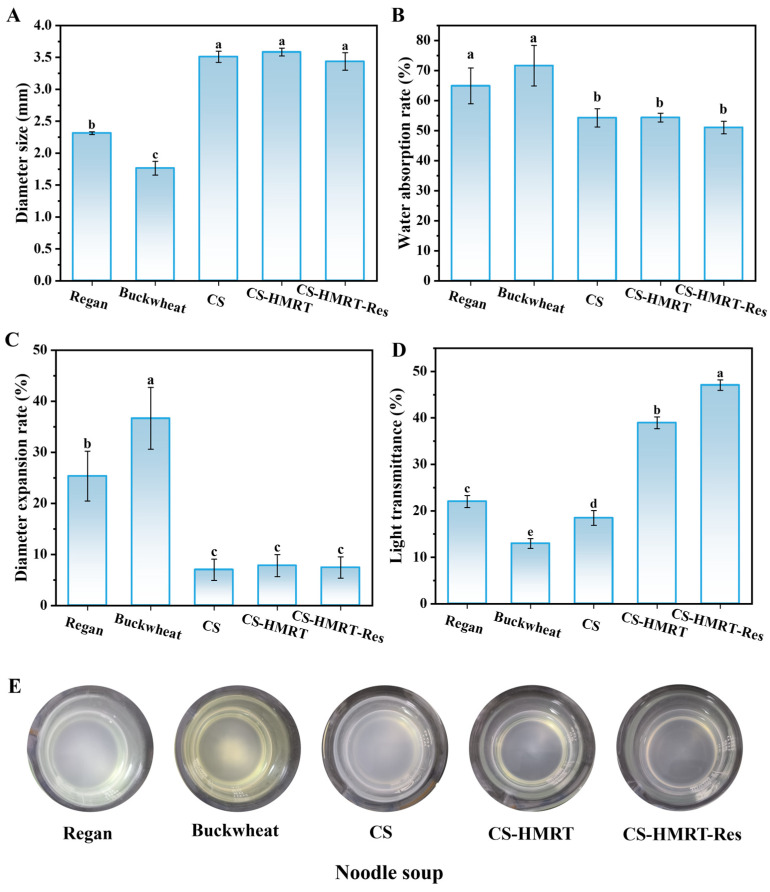
Quality attributes of noodles. Diameter (**A**), water absorption (**B**), diameter expansion (**C**), cooking loss (**D**), images of cooking liquor (**E**). Lowercase letters represent significant differences between different groups.

Immediately after exiting the die, the extruded CS-based noodles (CS, CS-HMRT and CS-HMRT-Res) expanded as internal pressure was released, producing a noodle diameter of ~3.5 mm. By contrast, commercial Regan noodles and buckwheat noodles exhibited smaller diameters (~2.3 mm and ~1.7 mm, respectively; Figure 1A). All samples met the minimum industrial requirement for water absorption (>15%) according to T/HBLS 0018-2023 [24]. Water absorption followed the order Buckwheat noodles > Regan noodles > CS-based extruded noodles (Figure 1B). The comparatively lower water uptake of extruded CS-based noodles may be associated with their more compact structure formed under thermo-mechanical processing, which could limit subsequent swelling during cooking. Notably, CS-HMRT-Res exhibited the lowest absorption, plausibly due to hydrophobic interactions and additional noncovalent crosslinking introduced by Res that reduced water accessibility.

Diameter expansion during cooking was highest in buckwheat noodles (~35%) and Regan noodles (~25%). Extruded CS-based noodles expanded substantially less (Figure 1C), indicating enhanced dimensional stability. Cooking loss, assessed via cooking-liquor transmittance [25], showed that CS-HMRT-Res generated the clearest broth (highest transmittance; Figure 1D,E), demonstrating the lowest leaching of soluble solids. Together, these results suggest that extrusion, especially when combined with HMRT-CS–Res, promoted a stronger continuous phase (starch–protein–polyphenol) that restricted starch dissolution and suppressed excessive swelling—two primary drivers of cooking loss.

**Figure 2:**
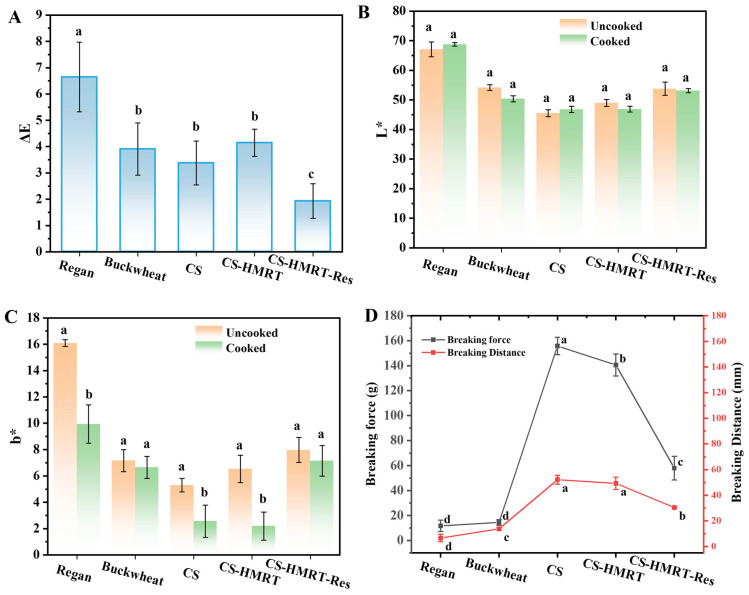
Total color difference after cooking (ΔE, (**A**)), lightness (L*, (**B**)), yellowness (b*, (**C**)) and tensile strength (**D**). Lowercase letters represent significant differences between uncooked and cooked noodles in (**B**,**C**). Lowercase letters represent significant differences between different groups in (**A**–**D**).

Color stability is also important for consumer acceptance. Regan noodles exhibited the largest total color difference after cooking (ΔE = 6.5; Figure 2A), whereas CS-HMRT-Res showed markedly improved color stability (ΔE = 1.8). Lightness (L*) changed minimally across groups (Figure 2B). In contrast, yellowness (b*, Figure 2C) was more sensitive to formulation and cooking treatment than L*. The higher change in b* observed in Regan noodles after cooking may be associated with greater solid leaching and a looser surface matrix, whereas the relatively stable b* value of CS-HMRT-Res suggests improved retention of color-related components during cooking. In addition, thermal hydration and surface restructuring during cooking may further contribute to the differences in yellowness among raw and cooked noodles.

#### 3.1.2. Texture Characteristics

Tensile testing provides an integrated measure of strand integrity and chewiness. Extruded CS-based noodles required a higher maximum tensile force than Regan and buckwheat noodles (Figure 2D), indicating a mechanically reinforced structure. Break distance followed a similar trend, with CS showing the highest extensibility (~45 mm), followed by CS-HMRT (~42 mm) and CS-HMRT-Res (~39 mm). Although adding Res slightly reduced extensibility relative to CS alone, the CS-HMRT-Res strands still outperformed commercial references, suggesting that polyphenol-mediated interactions increased cohesive strength while modestly limiting chain mobility.

The textural differences among noodle samples were closely related to the compactness and continuity of their internal networks. Table 1 showed that hardness, gumminess, and chewiness decreased in the order CS > CS-HMRT > CS-HMRT-Res > Regan noodles > buckwheat noodles. The markedly higher hardness, gumminess, and chewiness of the CS-based extruded noodles suggest that extrusion promoted the formation of a dense starch-rich continuous phase, thereby increasing resistance to compression and mastication. Notably, CS exhibited the highest hardness-related parameters, indicating that the untreated chestnut starch system formed the most rigid matrix after extrusion. By comparison, HMRT treatment slightly reduced these values, possibly because starch chain rearrangement during heat-moisture treatment moderated excessive rigidity while maintaining structural coherence [26]. After resveratrol incorporation, CS-HMRT-Res still exhibited higher springiness and chewiness than the commercial controls (Regan and Buckwheat noodles), but its hardness decreased relative to CS, suggesting that resveratrol did not simply strengthen the matrix, but rather adjusted intermolecular interactions to produce a more balanced texture. A similar trend has been reported in recent studies. Wang et al. [27] found that moderate addition of yellow onion skin extract improved cooked noodle hardness, chewiness, tensile strength, and elongation, which was associated with a denser gluten network, whereas excessive addition caused discontinuous structures and impaired quality. Likewise, Liu et al. [28] reported that the incorporation of konjac glucomannan and curdlan into extruded rice pasta increased hardness, chewiness, springiness, cohesiveness, and resilience, and these improvements corresponded to a more compact and continuous network structure. Therefore, the superior overall texture of CS-HMRT-Res in the present study was likely attributable to a more integrated and better-regulated matrix, rather than simply to maximal hardness.

#### 3.1.3. Flavor and Sensory Evaluation

**Figure 3:**
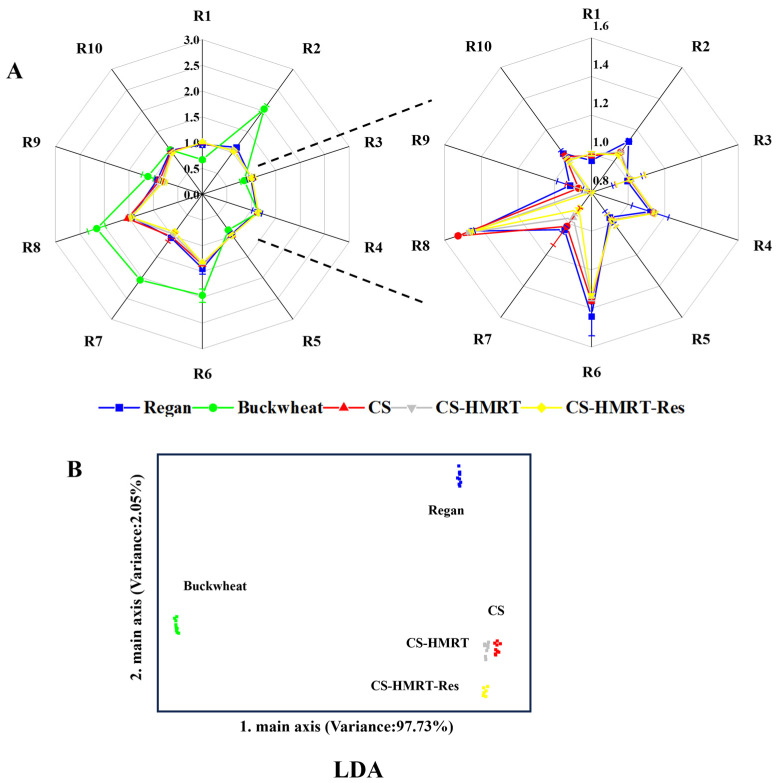
Electronic nose sensor response intensity (**A**) and linear discriminant analysis plot (**B**). Note: Ten sensors were used to detect aromatic compounds containing benzene (R1), nitrogen oxides (R2), ammonia compounds and aromatic compounds (R3), hydrogen compounds (R4), short-chain alkanes (R5), methyl compounds (R6), inorganic sulfides (R7), alcohols and aldehydes/ketones (R8), aromatic compounds and organic sulfides (R9), and long-chain alkanes (R10).

Flavor was considered a fundamental quality attribute influencing consumer preference for noodle products [29]. Electronic nose responses indicated that the overall volatile intensity of Regan noodles and CS-based noodles was comparable (*p* > 0.05; Figure 3A). Buckwheat noodles showed stronger signals for several sensors associated with nitrogen oxides (R2), methyl compounds (R6), inorganic sulfides (R7), and alcohols and aldehydes/ketones (R8). Linear discriminant analysis separated samples with a cumulative contribution of 97.73% (Figure 3B), clustering CS-based noodles apart from both Regan noodles and buckwheat noodles. These results demonstrate that substituting with chestnut starch creates a characteristic aroma fingerprint, while Res did not dominate the headspace profile.

**Figure 4:**
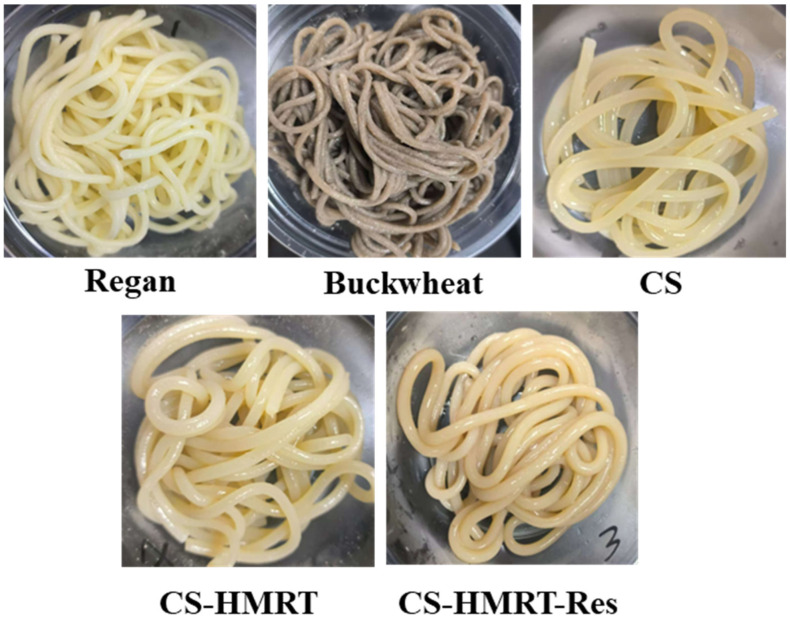
Photographs of noodles.

Sensory scores (Table 2, Figure 4) broadly agreed with instrumental measurements. CS-HMRT-Res did not differ significantly from Regan noodles in color and visual appearance (*p* > 0.05), while CS-HMRT and CS-HMRT-Res showed comparable surface smoothness to the reference. Overall acceptability ranked CS > CS-HMRT ≈ CS-HMRT-Res > Regan noodles > buckwheat noodles. Nonetheless, CS-HMRT-Res received lower scores for oral comfort and taste/aroma, which may reflect mild astringency associated with polyphenols. Future formulation work could mitigate this by optimizing Res dosage, encapsulation efficiency, or co-ingredients that mask astringency without compromising the low-GI effect.

### 3.2. Structural Analysis

#### 3.2.1. Microstructure

**Figure 5:**
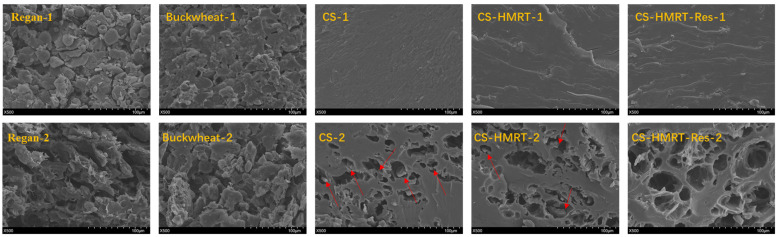
SEM micrographs of noodles: (500×) of uncooked (1) and cooked (2) samples; red arrows indicate dispersed granular solids.

SEM images revealed clear formulation-dependent differences in noodle microstructure before and after cooking (Figure 5). Uncooked Regan noodles exhibited a relatively loose matrix with discernible starch granules [30], and buckwheat noodles appeared denser with more agglomerated granules, whereas extruded CS-based noodles displayed a smoother, more compact surface with shear-induced striations, reflecting extrusion-induced structural consolidation. After cooking, all samples developed pores attributable to water-channel formation during gelatinization [31], but their morphology differed substantially. Regan noodles displayed severe fragmentation and sheet-like porous regions, while CS and CS-HMRT still contained large voids with residual granular solids, suggesting incomplete incorporation of granules into the continuous phase. In contrast, CS-HMRT-Res showed a more homogeneous porous structure without obvious dispersed granules, indicating a better-integrated matrix and improved starch retention during cooking. This result is consistent with its lower water absorption and cooking loss. Recent studies similarly showed that appropriate polyphenol addition can densify noodle networks and improve cooking stability, whereas excessive levels may induce structural discontinuity [27]; enhanced interpenetrating networks in extruded starch-based noodles have also been reported to improve structural integrity during processing [32]. Therefore, the superior microstructure of CS-HMRT-Res likely reflects a synergistic effect of extrusion-induced matrix formation and resveratrol-mediated intercomponent interactions, which together improved strand cohesion during cooking.

#### 3.2.2. Short-Range Ordered Structure

**Figure 6:**
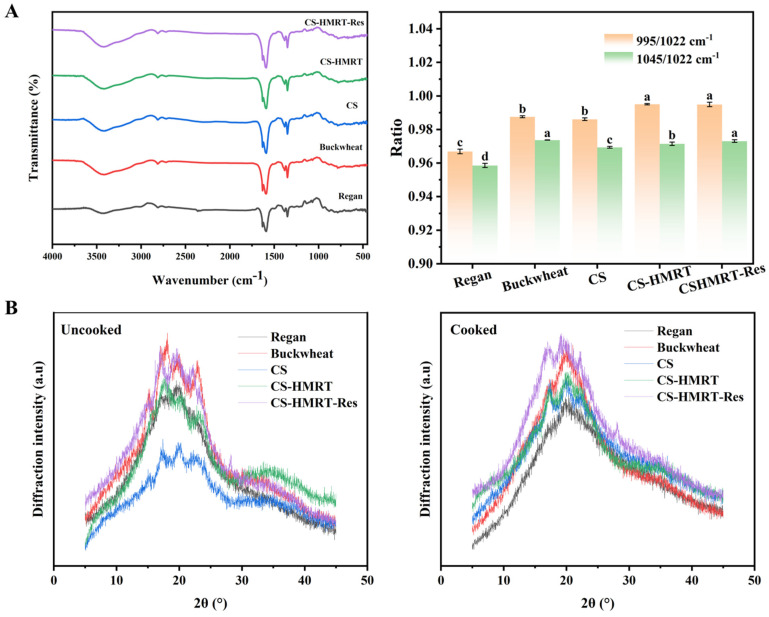
(**A**) short-range ordered structure and (**B**) long-range ordered structure. Lowercase letters represent significant differences between different groups.

FTIR was used to probe short-range ordering, conformational changes, and functional groups [33]. FTIR spectra were broadly similar across samples (Figure 6A), indicating that Res did not introduce new covalent functional groups and that interactions were primarily noncovalent, consistent with Zhao et al. [34], who observed no new peaks upon ferulic acid addition. All samples displayed characteristic peaks at 3400 cm^−1^ and 2950 cm^−1^, corresponding to the O–H stretching vibrations of hydroxyl groups [35]. To quantify short-range order, intensity ratios associated with ordered helical conformations (1045 cm^−1^ and 995 cm^−1^) relative to amorphous regions (1022 cm^−1^) were evaluated [36,37]. CS-based noodles exhibited higher R995/1022 and slightly higher R1045/1022 than the Regan noodle reference, suggesting increased double-helical content and local molecular ordering. These changes are consistent with extrusion-driven chain alignment and HMRT-induced recrystallization. The modest increase observed for CS-HMRT-Res supports the hypothesis that Res facilitates chain aggregation and stabilization of ordered motifs, likely via hydrogen bonding and hydrophobic stacking.

#### 3.2.3. Long-Range Ordered Structure

XRD patterns (Figure 6B) further supported processing-induced reorganization [38]. Regan noodles typically exhibit A-type diffraction peaks (2θ ≈ 15°, 17°, 18°, and 23°), which largely disappeared after cooking, indicating disruption of native crystallites [39]. All cooked noodle samples displayed a characteristic V-type peak near 20° [11], commonly attributed to single-helical inclusion complexes (e.g., amylose–lipid/protein/polyphenol) [40]. CS-HMRT-Res also showed a peak consistent with Res crystalline near 27°, indicating that a fraction of Res remained within the matrix.

Relative crystallinity of cooked noodles increased from Regan noodles (~25.8%) to CS-HMRT-Res (~30.8%), with CS and CS-HMRT intermediate. Higher crystallinity generally reflects a more ordered internal structure that can restrict enzyme penetration and hydrolysis. In the present system, the combination of extrusion and HMRT likely promoted amylose release and subsequent V-type complexation and reordering during cooling [41], while Res further stabilized the compact arrangement. These results are consistent with the FTIR-derived increases in short-range order.

### 3.3. In Vitro Digestibility and In Vivo Glycemic Response

**Figure 7:**
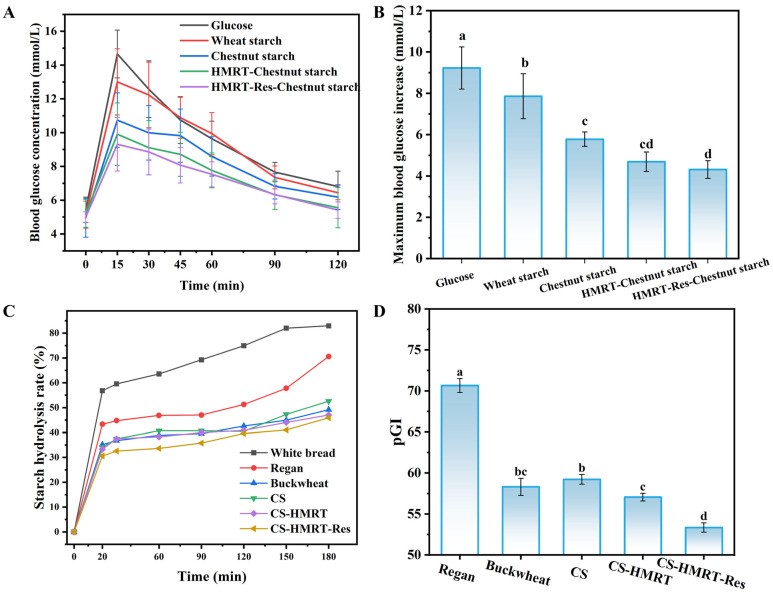
Glycemic response and digestion: (**A**) postprandial blood glucose curves in mice; (**B**) maximum blood glucose increments; (**C**) in vitro starch hydrolysis profiles; (**D**) predicted glycemic index. Lowercase letters represent significant differences between different groups.

Mice across groups exhibited similar fasting glucose (4.9–5.3 mmol/L), indicating comparable baseline metabolism. Following gavage, the glucose reference produced the expected rapid spike (peak 14.66 mmol/L at 15 min; Figure 7A). Starch groups also peaked at 15 min but with substantially lower maxima. At 120 min, mice receiving HMRT-Chestnut starch and HMRT-Res-Chestnut starch remained significantly lower than the glucose group, indicating faster recovery toward baseline. The maximum increment in blood glucose was lowest for HMRT-Res-Chestnut starch (4.31 mmol/L), followed by HMRT-Chestnut starch and untreated Chestnut starch, and was highest for wheat starch (7.86 mmol/L; Figure 7B). These results demonstrate that chestnut starch inherently elicited a lower glycemic response than wheat starch and that HMRT and Res complexation further amplified this benefit [42].

In vitro starch digestion curves were commonly employed to simulate in vivo digestion processes and to predict the glycemic index (pGI) [43]. The pGI reflects the capacity of carbohydrate-rich foods to raise blood glucose relative to glucose or white bread [44]. In vitro digestion profiles paralleled the in vivo trends. Final hydrolysis at 180 min decreased from white bread (82.96%) and Regan noodles (70.58%) to CS-based noodles, with the lowest value in CS-HMRT-Res (45.92%; Figure 7C). Calculated pGI values ranked Regan noodles (70.65; high-GI) > CS (59.23) ≈ buckwheat (58.31) ≈ CS-HMRT (57.06) > CS-HMRT-Res (53.35; low-GI; Figure 7D). Mechanistically, the reduced hydrolysis of CS-HMRT-Res can be attributed to synergistic effects: (i) increased molecular order and V-type complex formation that physically limits enzyme access [45], (ii) competitive binding of Res to α-amylase, and (iii) altered hydration/swelling behavior that slows diffusion of enzymes and hydrolysis products. Together, these findings support the feasibility of designing extrusion-manufactured fresh noodles with a low predicted glycemic impact.

Overall, these findings demonstrate that HMRT-Res could reduce noodle digestibility and slow the sharp rise in postprandial blood glucose, indicating its potential in lowering the incidence of diabetes, obesity, and cardiovascular diseases. To further clarify the interaction mechanism among Res, starch chains and α-amylase, molecular dynamics simulation was carried out in the subsequent study, offering a theoretical foundation for the functional application of polyphenols in low-glycemic-index foods.

### 3.4. Molecular Dynamics Insights into α-Amylase Inhibition

**Figure 8:**
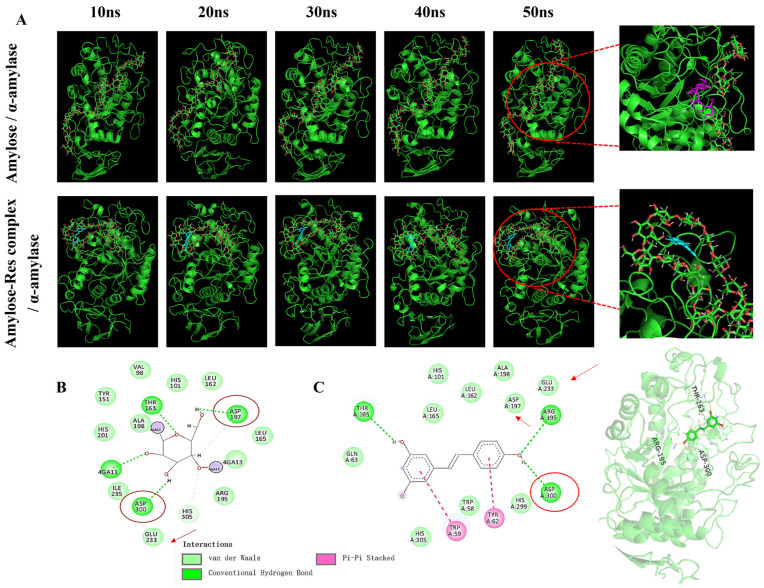
Molecular dynamics simulations: (**A**) representative binding conformations of amylose–amylase and amylose–Res–amylase systems; (**B**) noncovalent interactions between glucose residues and α-amylase; (**C**) noncovalent interactions between Res and α-amylase. α-Amylase structure was obtained from PDB ID 1B2Y.

The catalytic region of α-amylase comprises key residues Glu233, Asp197, and Asp300, which are essential for substrate recognition and hydrolysis [46,47,48]. To further explain the lower in vitro starch hydrolysis and reduced pGI observed for the CS-HMRT-Res system, MD simulations were used to probe how Res may interfere with starch recognition and catalysis by α-amylase. In the amylose–amylase system, the starch chain progressively adopted conformations that enabled entry into the enzyme binding cavity, forming hydrogen bonds with catalytic residues (Asp197 and Asp300) and stabilizing contacts near Glu233 (Figure 8A,B). When Res was present, the amylose–Res complex showed reduced penetration into the cavity, suggesting that Res altered chain conformation and/or sterically hindered productive binding [6]. Direct interaction analysis further indicated that Res preferentially occupied the active-site region through a combination of van der Waals forces, hydrogen bonding, and π-π interactions with aromatic residues (Figure 8C). This comparison suggests that resveratrol not only competes with starch for access to the catalytic region, but also perturbs starch conformation in a way that reduces its enzymatic susceptibility. Combined with the higher molecular order and enhanced V-type complexation observed experimentally, these results provide a coherent mechanistic explanation for the lower hydrolysis extent and glycemic response of CS-HMRT-Res noodles.

## 4. Conclusions

Extruded fresh noodles fortified with CS-HMRT-Res complexes were successfully produced and systematically characterized. CS-HMRT-Res incorporation improved processing quality by decreasing water uptake, limiting diameter expansion, and reducing cooking loss, while maintaining desirable sensory attributes. Structural analyses (SEM, FTIR, and XRD) indicated that CS-HMRT-Res promoted a more compact and ordered starch network with enhanced V-type complexation and increased crystallinity after cooking. Functionally, CS-HMRT-Res reduced in vitro starch hydrolysis and achieved a low pGI (53.35), and attenuated postprandial blood glucose excursions in mice. MD simulations suggested that Res can occupy the α-amylase binding cavity and restrict starch accessibility, providing a mechanistic rationale for enzyme inhibition and delayed digestion. From an application perspective, extrusion-enabled CS-HMRT-Res fortification provides a promising strategy for developing fresh noodle products with improved structural stability and reduced glycemic potential. Such low-GI staple foods may have strong market potential for consumers seeking healthier carbohydrate-based diets, particularly those concerned with postprandial glycemic control.

## Figures and Tables

**Table 1:** Textural characteristics of cooked noodles.

Samples	Hardness (g)	Springiness	Cohesiveness	Gumminess	Chewiness	Resilience
Regan	1584.01 ^cd^ ± 74.15	0.84 ^b^ ± 0.03	0.81 ^bc^ ± 0.04	1289.58 ^cd^ ± 107.74	1073.84 ^c^ ± 61.88	0.61 ^b^ ± 0.08
Buckwheat	982.79 ^d^ ± 62.19	0.85 ^b^ ± 0.02	0.80 ^c^ ± 0.01	785.87 ^d^ ± 47.95	664.66 ^c^ ± 36.36	0.60 ^b^ ± 0.02
CS	3499.78 ^a^ ± 486.34	0.97 ^a^ ± 0.03	0.95 ^a^ ± 0.01	3311.37 ^a^ ± 478.5	3175.55 ^a^ ± 370.27	0.76 ^a^ ± 0.01
CS-HMRT	2914.81 ^ab^ ± 150.42	0.95 ^a^ ± 0.03	0.88 ^ab^ ± 0.03	2555.54 ^ab^ ± 87.36	2416.10 ^b^ ± 96.32	0.72 ^ab^ ± 0.05
CS-HMRT-Res	2221.49 ^bc^ ± 266.26	0.98 ^a^ ± 0.03	0.85 ^bc^ ± 0.02	1887.74 ^bc^ ± 204.87	1844.90 ^b^ ± 222.57	0.66 ^ab^ ± 0.02

Note: Data are presented as the mean ± SD, n = 3. Different superscript letters in the same column denote significant differences (Duncan’s range test, *p* < 0.05).

**Table 2:** Sensory analysis of cooked noodles.

Samples	Color (Points)	Visual Appearance (Points)	Oral Comfort (Points)	Viscoelasticity (Points)	Surface Smoothness (Points)	Taste & Aroma (Points)	Overall Score (Points)
Regan	16.83 ^ab^ ± 0.47	7.67 ^bc^ ± 0.28	15.83 ^a^ ± 0.99	20.75 ^b^ ± 1.14	11.25 ^bc^ ± 0.54	2.83 ^a^ ± 0.27	75.17 ^bc^ ± 2.80
Buckwheat	13.67 ^c^ ± 1.01	6.92 ^c^ ± 0.40	14.33 ^a^ ± 1.09	21.00 ^b^ ± 1.13	10.92 ^c^ ± 0.58	3.25 ^a^ ± 0.30	69.33 ^c^ ± 3.61
CS	17.50 ^a^ ± 0.44	8.75 ^a^ ± 0.28	10.92 ^b^ ± 1.10	28.67 ^a^ ± 0.33	13.92 ^a^ ± 0.31	3.33 ^a^ ± 0.22	83.08 ^a^ ± 1.44
CS-HMRT	16.67 ^ab^ ± 0.45	8.00 ^ab^ ± 0.30	10.50 ^b^ ± 1.12	28.92 ^a^ ± 0.45	12.92 ^ab^ ± 0.56	3.13 ^a^ ± 0.20	80.13 ^ab^ ± 1.75
CS-HMRT-Res	15.50 ^b^ ± 0.65	7.33 ^bc^ ± 0.36	10.17 ^b^ ± 0.72	28.00 ^a^ ± 0.43	12.67 ^ab^ ± 0.81	2.08 ^b^ ± 0.29	77.33 ^ab^ ± 2.24

Note: Data are presented as the mean ± SD, n = 20. Different superscript letters in the same column denote significant differences (Duncan’s range test, *p* < 0.05).

## Data Availability

The original contributions presented in this study are included in the article. Further inquiries can be directed to the corresponding author.
